# *Staphylococcus aureus* suppresses the pentose phosphate pathway in human neutrophils via the adenosine receptor A2aR to enhance intracellular survival

**DOI:** 10.1128/mbio.02571-23

**Published:** 2023-12-18

**Authors:** Emilio G. Vozza, Clíodhna M. Daly, Sinead A. O'Rourke, Hannah K. Fitzgerald, Aisling Dunne, Rachel M. McLoughlin

**Affiliations:** 1 Host-Pathogen Interactions Group, School of Biochemistry and Immunology, Trinity Biomedical Sciences Institute, Trinity College Dublin, Dublin, Ireland; 2 Molecular Immunology Group, School of Biochemistry and Immunology, Trinity College Dublin, Dublin, Ireland; Harvard Medical School, Boston, Massachusetts, USA; University of Nebraska Medical Center, Omaha, Nebraska, USA

**Keywords:** *Staphylococcus aureus*, intracellular pathogen, pentose phosphate pathway, metabolism, A2aR, *adora2a*, host-pathogen interactions, neutrophil

## Abstract

*Staphylococcus aureus* propensity for intracellular survival presents a challenge to effectively treat infection, with significant evidence suggesting *S. aureus* can hijack phagocytes for host dissemination, worsening clinical outcomes. Human polymorphonuclear neutrophils (hPMN) have been identified as an important conduit for the intracellular survival of *S. aureus,* yet this immune evasion mechanism remains incompletely understood. Multiplex gene expression analysis was used to profile the gene expression of hPMN harboring intracellular *S. aureus* and found that the anti-inflammatory adenosine receptor, *adora2a* (A2aR), was upregulated along with several A2aR- associated genes. Importantly, treatment with the A2aR inhibitor, ZM-241385, reduced intracellular survival of *S. aureus* within hPMN while enhancing ROS production and neutrophil extracellular traps. This suggests that *S. aureus* uses A2aR to dampen hPMN effector functions as a mechanism to boost intracellular survival. A2aR inhibition led to a metabolic switch from glycolysis to the pentose phosphate pathway (PPP) in hPMN, which facilitated an increase in NADPH levels and reactive oxygen species (ROS) production. Taken together, our work highlights the importance of the A2aR, which is targeted by *S. aureus* to suppress the PPP, limiting ROS production, and thus creating a more hospitable intracellular niche in which to survive.

**IMPORTANCE:**

*Staphylococcus aureus* is one of the leading causes of antimicrobial-resistant infections whose success as a pathogen is facilitated by its massive array of immune evasion tactics, including intracellular survival within critical immune cells such as neutrophils, the immune system’s first line of defense. In this study, we describe a novel pathway by which intracellular *S. aureus* can suppress the antimicrobial capabilities of human neutrophils by using the anti-inflammatory adenosine receptor, *adora2a* (A2aR). We show that signaling through A2aR suppresses the pentose phosphate pathway, a metabolic pathway used to fuel the antimicrobial NADPH oxidase complex that generates reactive oxygen species (ROS). As such, neutrophils show enhanced ROS production and reduced intracellular *S. aureus* when treated with an A2aR inhibitor. Taken together, we identify A2aR as a potential therapeutic target for combatting intracellular *S. aureus* infection.

## INTRODUCTION

*Staphylococcus aureus* is one of the most frequent causes of bloodstream infections with mortality rates ranging from 20% to 30% (1, 2). This is further complicated by the high prevalence of antimicrobial resistance*,* with methicillin-resistant *S. aureus* alone being associated with ~100,000 deaths worldwide in 2019 (3). Additionally, *S. aureus* possesses a wide array of immune evasion mechanisms that worsen disease severity and prolong infection. While long considered an extracellular bacterium, it is now appreciated that *S. aureus* is adept at surviving intracellularly (4), which provides a protective intracellular niche to shield *S. aureus* from both antibiotics and the humoral immune response (5). Intracellular survival within human phagocytes has been associated with increased minimum inhibitory concentrations of vancomycin (6, 7), while also being highlighted as a predominant feature among clinical isolates from *S. aureus* bacteremia, bone/joint infection, and infective endocarditis patients in a recent multiparametric study (8). Importantly, this creates bacterial reservoirs for recurrent infection allowing for host dissemination which worsens clinical outcome (9, 10).

Despite their importance in the protective immune response to *S. aureus*, polymorphonuclear neutrophils (PMN) can also serve as a reservoir for intracellular *S. aureus*, acting as a “Trojan horse,” creating a protective niche that can facilitate dissemination (11). The importance of PMN during intracellular survival has been shown in several murine models. PMN taken from the site of infection harboring intracellular *S. aureus* could establish infection when transferred to naive mice (12), while in a surgical wound model, reduced CXC chemokine expression and neutrophil accumulation was associated with an overall reduction in bacterial burden (13, 14). Additionally, *S. aureus* was shown to preferentially survive inside circulating PMN when compared to other phagocyte populations following intraperitoneal challenge (15). More recently, PMN depletion with anti-Ly6g antibodies restricted *S. aureus* to the liver only, following intravenous challenge, whereas mice, in which circulating PMN were unchanged, showed bacterial dissemination implicating PMN as vessels for host dissemination (9).

Intracellular *S. aureus* has been shown to manipulate host macroautophagy, a recycling pathway that uses double-membraned vesicles, termed autophagosomes, to transport damaged organelles and proteins to the lysosome (16). The accumulation of autophagosomes creates a protective intracellular niche in which the bacterium can viably grow and divide. Additionally, intracellular *S. aureus* uses crosstalk between autophagy and apoptosis pathways to prolong the human polymorphonuclear neutrophil (hPMN) life span and thus protect its intracellular niche. In a zebrafish model, *S. aureus* was shown to manipulate the non-canonical autophagy pathway known as LC3-associated phagocytosis as a mechanism for intracellular survival within spacious single membrane vesicles decorated with the autophagy protein LC3 (17).

While autophagy appears to be important in the intracellular lifestyle of *S. aureus* within PMN, little is known as to what other pathways may be involved or how they might contribute to intracellular survival. It is known that *S. aureus* can suppress protective inflammatory environments to facilitate its survival in multiple settings (18–22), but it is unclear whether intracellular *S. aureus* specifically promotes an immunosuppressive phenotype within hPMN. In this study, gene expression analysis revealed increased expression of the anti-inflammatory adenosine receptor, *adora2a* (A2aR), within hPMN harboring intracellular *S. aureus*. Intervention with an A2aR inhibitor led to significant reductions in intracellular burden, enhanced NETosis, and reactive oxygen species (ROS) production. Interestingly, inhibition of A2aR signaling diverted hPMN glycolysis to the pentose phosphate pathway (PPP) leading to enhanced ROS production. This study describes a novel mechanism by which intracellular *S. aureus* can restrict hPMN anti-microbial effector functions by inducing the adenosine-sensing A2aR to metabolically reprogram hPMN and restrict PPP, reducing NADPH levels and consequently ROS production.

## MATERIALS AND METHODS

### Bacterial cell culture

*S. aureus* strain LAC USA300 (23) was cultivated from frozen stocks streaked onto tryptic soy agar (TSA) plates and incubated overnight at 37°C. Bacterial inocula were prepared in phosphate-buffered saline (PBS) to 1 × 10^9^ CFU/mL using an OD_600_ of 1. Prior to infection, bacteria were opsonized using human intravenous immunoglobulin (IVIG, 5 mg/mL, Kiovig) and guinea pig complement (Cedarlane) for 20 min at 37°C to facilitate opsonophagocytic uptake by hPMN.

### Human neutrophil isolation

Fresh peripheral blood was collected from healthy donors into lithium heparin collection tubes after informed consent under a protocol that was approved by the Trinity College Dublin Faculty of Science, Technology, Engineering, and Mathematics research ethics committee. Neutrophils were isolated by dextran sedimentation and gradient separation using Lymphoprep (Axis shield, Progen). Red blood cells were lysed using ammonium–chloride–potassium (ACK) lysis buffer (Gibco), and the resulting hPMN was resuspended in Dulbecco’s Modified Eagle’s Medium with Glutamax (Gibco) supplemented with 10% fetal bovine serum (Sigma) and HEPES (10 µM, Sigma). Unless otherwise stated, the final concentration of hPMN was 2 × 10^6^ cells per replicate. Cell purity was assessed by flow cytometry using the neutrophil markers CD15 and CD16 which was confirmed to be >90%.

### Intracellular survival assay

*S. aureus* intracellular survival assays were conducted as previously described (16). Briefly, hPMN were infected for 1 h with pre-opsonized *S. aureus* (MOI 10) under rotation at 37°C followed by the addition of gentamicin (100 µg/mL, Sigma) to eliminate extracellular bacteria. The addition of gentamicin was designated 0 h. Some groups were pretreated with ZM-241385 (10 µM, Sigma), CGS 21680 (1 µM), 6-aminonicotinamide (500 µM), or corresponding vehicle controls 30 min prior to infection and showed no cytotoxicity in response to treatment. At specific time points, hPMN were lysed with Triton X-100 (0.1%, Sigma), diluted with PBS (Sigma), and plated on TSA plates for CFU enumeration.

### RNA isolation, cDNA synthesis, and RT-PCR

Total RNA was isolated using the Qiagen miRNeasy kit according to the manufacturer’s protocol. RNA yield and quality were assessed using a Spectrostar Nano spectrophotometer and LVIS plate. RNA (250 ng) was reverse-transcribed using a High-Capacity cDNA Reverse Transcription Kit (Applied Biosystems) according to the manufacturer’s instructions. RT-PCR was conducted using a CFX96 Touch Real-Time PCR Detection System (Bio-Rad) using iTaq Sybr Green Supermix (Bio-Rad) according to the manufacturer’s instructions. The human-specific primer used for *adora2a* was obtained from Integrated DNA Technologies (Hs.PT.56a.20721611.g). Gene expression was normalized to *actb* and analyzed using the ΔΔCT method.

### Nanostring gene expression analysis

RNA was isolated from hPMN harboring intracellular *S. aureus* at 3 h post gentamicin treatment and assessed using the human myeloid panel as per the manufacturer’s protocol using the nCounter platform (Nanostring technologies). Gene expression analysis was conducted using the software program nSolver with the advanced analysis plug-in. Fold change was calculated relative to uninfected hPMN control. Statistically, genes are compared using a Student’s *t*-test, comparing the sample mean to the baseline mean of an uninfected hPMN control. To account for false discoveries, the Benjamini-Yekutieli post-test procedure was performed to determine the false discovery rate adjusted *P* value of each target gene.

### Flow cytometry

hPMN were centrifuged at 300 g for 5 min, washed with PBS, and incubated for 15 min with Zombie near IR fixable viability dye followed by 15-min incubation with an Fc receptor binding inhibitor polyclonal antibody (eBioscience). Cells were washed in PBS containing 1% bovine serum albumin. Cells were stained with a human A2aR Alexa Fluor 488-conjugated antibody (Clone #599717) or CD39 PE-conjugated antibody [eBioA1 (A1)] for 15 min and fixed with Fix & Perm fix A (Thermofisher) for 15 min. Samples were assessed using a BD FACSCanto II (BD biosciences) cytometer.

To measure ROS, hPMN were infected as above, and at 2.5 h post-gentamicin treatment, they were incubated with the cell-permeable ROS sensor CellRox green reagent for 30 min and treated as above.

### ELISA

At 6 h post-gentamicin treatment, cell supernatants were harvested and IL-8 (ELISA Max Deluxe Set Human IL-8, Biolegend), IL-6 (ELISA Max Standard Set Human IL-6, Biolegend), and MPO (Human Myeloperoxidase DuoSet ELISA, R&D systems) levels quantfied following the manufacturer’s guidelines.

### NETosis assay

To measure NETosis, the Abcam NETosis assay was employed, which uses net-associated neutrophil elastase (NE) activity as a surrogate marker. Briefly, 2 × 10^6^ hPMN/well were plated in a 24-well plate with some groups pre-treated with ZM-241385 for 30 min. hPMN were infected as above and 2 h post-gentamicin treatment, they were assayed as per the manufacturer’s guidelines using a Spectrostar Nano microplate reader.

### Seahorse glyco stress test

hPMN were seeded in a 96-well plate (5 × 10^5^ hPMN/well) and left to adhere for 30 min. hPMN were pretreated with dimethyl sulfoxide (DMSO) or ZM-241385 for 30 min and then stimulated with heat-killed *S. aureus* (HKSA) (2 µg/mL) for 2 h. hPMN were then centrifuged for 5 min at 300 *g* and resuspended in XF DMEM medium, pH 7.4, supplemented with 2 mM L-glutamine and 1 mM sodium pyruvate. hPMN were transferred to a Seahorse 96-well plate pre-coated with Cell Tak (Corning) as per the manufacturer’s guidelines and centrifuged at 200 *g* for 1 min without the centrifuge break. Finally, hPMN were incubated in a non-CO_2_ incubator before placing into an XF/XFe analyzer to measure the extracellular acidification (ECAR) during sequential injection of glucose (10 mM), oligomycin (1 µM), and finally 2-deoxyglucose (2DG) (50 mM). Analysis was conducted using Wave software analysis (Agilent Technologies), and basal glycolysis rate was calculated using the average ECAR values before the addition of oligomycin minus the non-glycolytic ECAR after 2DG addition.

### Lactate measurement

One hour post-gentamicin treatment, hPMN were lysed in triton X-100 (0.2%) for 10 min and assayed using the Lactate Assay Kit (Merck) as per the manufacturer’s guidelines.

### NADP/NADPH quantification

One hour post-gentamicin treatment, NADP/NADPH levels were assessed using the NADP/NADPH quantitation kit, which first converts NADP into NADPH to then quantify total NADPH (NADP + NAPDH) (MAK038-1KT, Merck). Briefly, pooled hPMN replicates (1 × 10^7^ hPMN) were extracted for 20 min in 400 µL extraction buffer and centrifuged at 10,000 *g* for 10 min. Extracted supernatants were centrifuged at 12,000 *g* for 20 min using a 10 kDa cutoff micron spin filter to deproteinize the samples. Samples were assayed as per the manufacturer’s guidelines, converting NADP to NADPH and assessing the combined level of NADP/NADPH.

### Western blotting

hPMN (6 × 10^6^ cells) were lysed using 1× SDS sample buffer and heated to 95°C for 5 min. The samples were separated using SDS-PAGE, transferred onto polyvinylidene difluoride membranes, and blocked using AdvanBlock (Advansta) for 1 h. The membranes were incubated overnight with rabbit anti-human G6PD antibody [Cell Signaling Technology (CST), 8866S] followed by an HRP-conjugated anti-rabbit secondary antibody (CST, 7074) for 1 h and imaged on a BioRad Chemidoc CCD imaging device with enhanced chemiluminescence (ECL) detection and ImageLab software analysis. Additionally, mouse anti-human β-actin (Sigma) was used as a loading control.

### Statistical analysis

Statistical analysis was performed using nSolver for the Nanostring analysis as described above and otherwise with Prism GraphPad 9 using a Wilcoxon’s test for direct comparison, and a one-way or two-way ANOVA for multiple comparisons using Dunn’s or Šídák’s post-test, respectively.

## RESULTS

### Pathway analysis of hPMN harboring *S. aureus* intracellular reveals significant changes in cytokine and growth factor signaling and highlights the upregulation of the anti-inflammatory Adora2a receptor

To understand the impact that intracellular *S. aureus* has on infected hPMN, gene expression analysis was conducted using RNA isolated from hPMN harboring intracellular *S. aureus*. hPMN were infected for 1 h with *S. aureus* strain LAC USA300 followed by gentamicin treatment to eliminate extracellular *S. aureus* and lysed at 3 h post-gentamicin treatment for RNA isolation. RNA was assessed using the Nanostring nCounter platform and the human myeloid panel that can detect up to 770 myeloid-associated genes. Of these, 448 differentially expressed genes (DEGs) from uninfected control (156 < FDR *P* value) were detected and used to perform pathway analysis. This was achieved using the Nanostring nSolver software, which groups genes into gene sets and uses each gene set’s first principal component to create a pathway score to identify increased gene expression.

**Fig 1:**
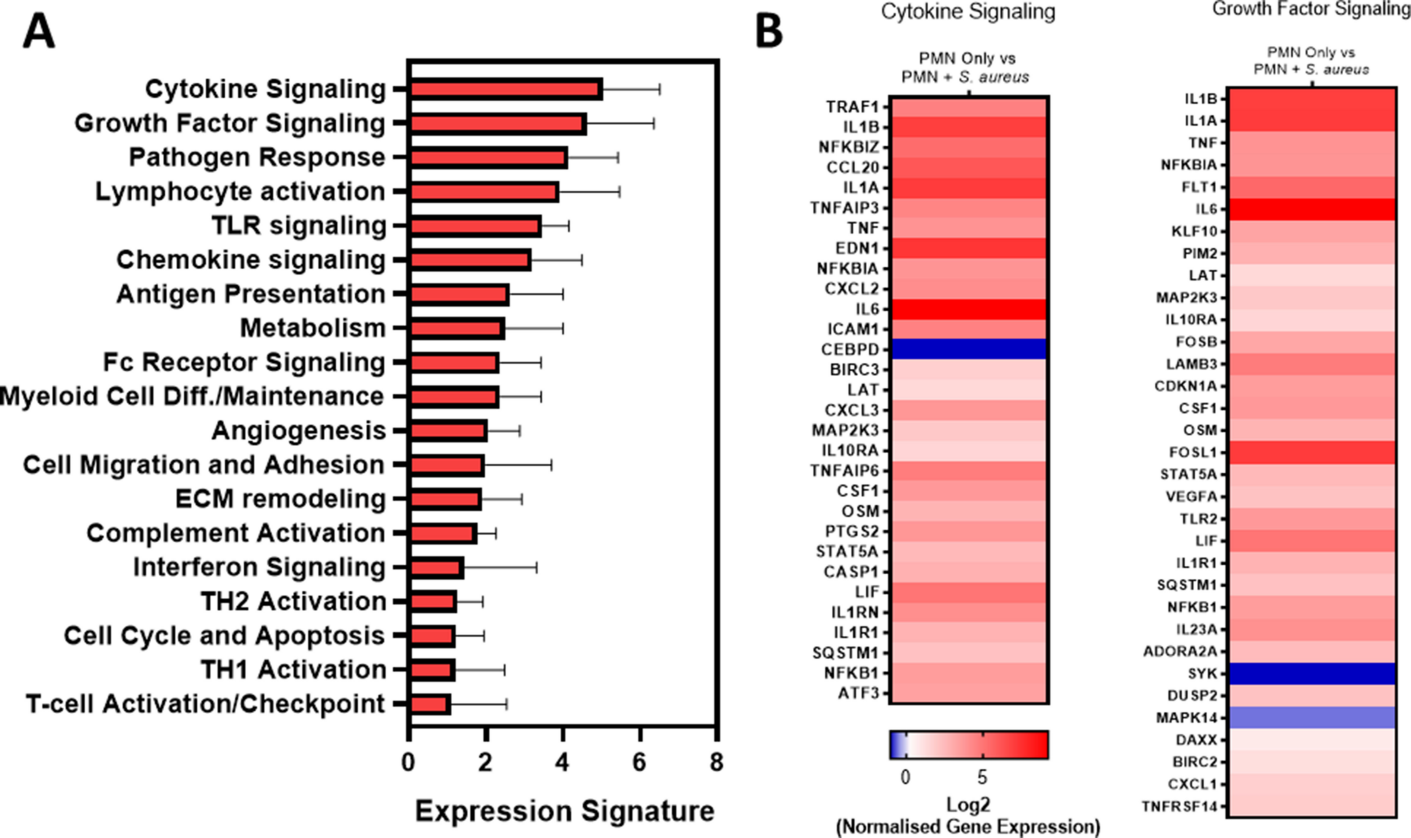
Intracellular *S. aureus* induces significant upregulation of several immune cell signaling pathways. RNA was isolated from hPMN harboring intracellular *S. aureus* 3 h post-gentamicin treatment and assessed using the Nanostring myeloid panel. Pathway analysis reveals that intracellular *S. aureus* activates multiple PMN pathways relative to uninfected PMN with cytokine and growth factor signaling showing the greatest induction (**A**). Heatmaps highlight the upregulation of several pro-inflammatory and anti-apoptotic genes as well as the autophagy marker p62 in hPMN harboring intracellular *S. aureus* (**B**). Pathway analysis clusters genes and uses each gene’s first principal component to create a pathway score. Gene expression represented in the heatmap analysis is measured relative to uninfected control using a Student’s *t* test with each gene having an FDR adjusted *P* value < 0.05 for *n* = 4 independent donors.

Pathway analysis revealed that the greatest pathway scores were associated with cytokine and growth factor signaling gene sets (Fig. 1A). Within these gene sets, hPMN harboring intracellular *S. aureus* were shown to express high levels of pro-inflammatory cytokines (*il1a*, *il1b*, and *il6*), chemokines (*cxcl1*, *cxcl2*, and *cxcl3*), and downstream signaling molecules (*traf1* and *nfkb1*) (Fig. 1B). Interestingly, the autophagy-related *sqstm1* (p62) was also significantly upregulated in hPMN harboring intracellular *S. aureus* in addition to several anti-apoptotic factors (*birc2*, *birc3*, and *tnfaip3*) and the p53-inducible gene *cdkn1a* (Fig. 1B). This is consistent with a previous study that showed intracellular *S. aureus* can induce an anti-apoptotic phenotype leading to p53-induced autophagy via DRAM1 (16).

**Fig 2:**
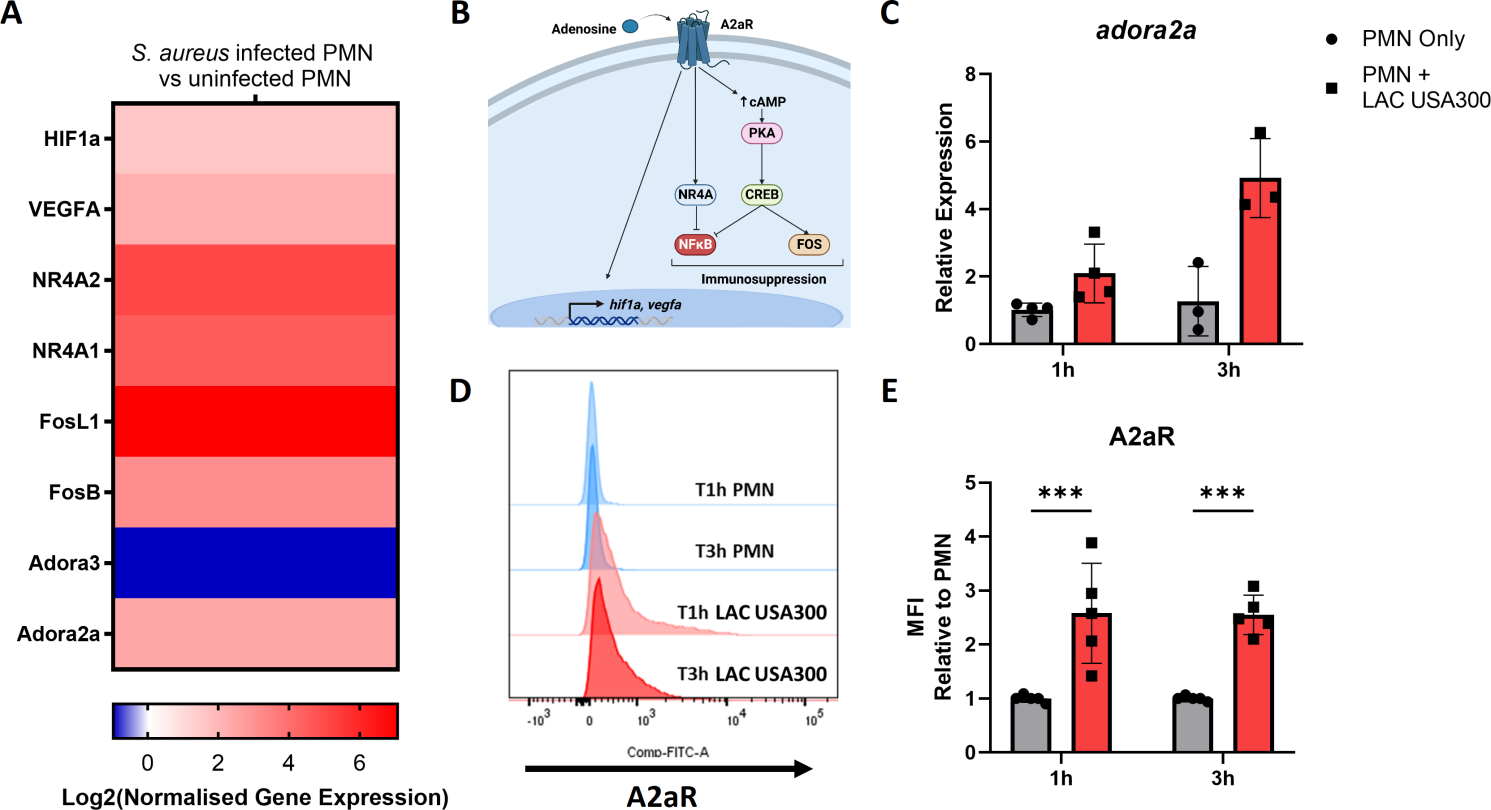
hPMN harboring intracellular *S. aureus* significantly upregulate A2aR expression as well as several A2aR-associated signaling molecules and genes. Nanostring gene expression analysis with an FDR adjusted *P* value < 0.05 highlights hPMN harboring intracellular *S. aureus* significantly upregulate A2aR as well as several A2aR-associated signaling molecules and genes (**A**). Schematic view of signaling molecules and proteins associated with A2aR activation (**B**). hPMN were infected with *S. aureus* for 1 h prior to gentamicin treatment and assessed using RT-PCR and flow cytometry. RT-PCR was conducted using the ΔΔCT method and normalized using *actB* as a housekeeping gene (**C**). Flow cytometric assessment of A2aR was conducted by gating on single, live cells to obtain representative histograms (**D**) and median fluorescence intensity (MFI) relative to uninfected hPMN (**E**). Statistical analysis was conducted using a two-way ANOVA with Šídák’s post-test (***P < 0.001) for *n* = 3–4 independent donors.

Pathway analysis of DEGs from hPMN harboring intracellular *S. aureus* also highlighted the anti-inflammatory adenosine receptor *adora2a* (Fig. 1B). Further analysis of DEGs showed several upregulated genes associated with A2aR (Fig. 2A). Such genes included *fosb*, *fosl*, *nr4a1*, and *nr4a2,* which contribute to A2aR-induced immunosuppression. A2aR acts through cAMP signaling and PKA to inhibit NF-κB via NR4a and the FOS family proteins (24–26) (Fig. 2B). Furthermore, upregulation of *hif1a*, which boosts adenosine-producing enzymes, and *vegfa*, an angiogenic factor associated with A2aR signaling (27, 28), was also observed. A2aR expression by hPMN harboring intracellular *S. aureus* was verified by RT-PCR and flow cytometry, which highlighted significant upregulation of A2aR at both the genes (Fig. 2C) and protein level (Fig. 2D and E). Additionally, hPMN were shown to upregulate A2aR in response to HKSA (Fig. S1).

### Intracellular survival of *S. aureus* in hPMN is significantly reduced by A2aR inhibition

**Fig 3:**
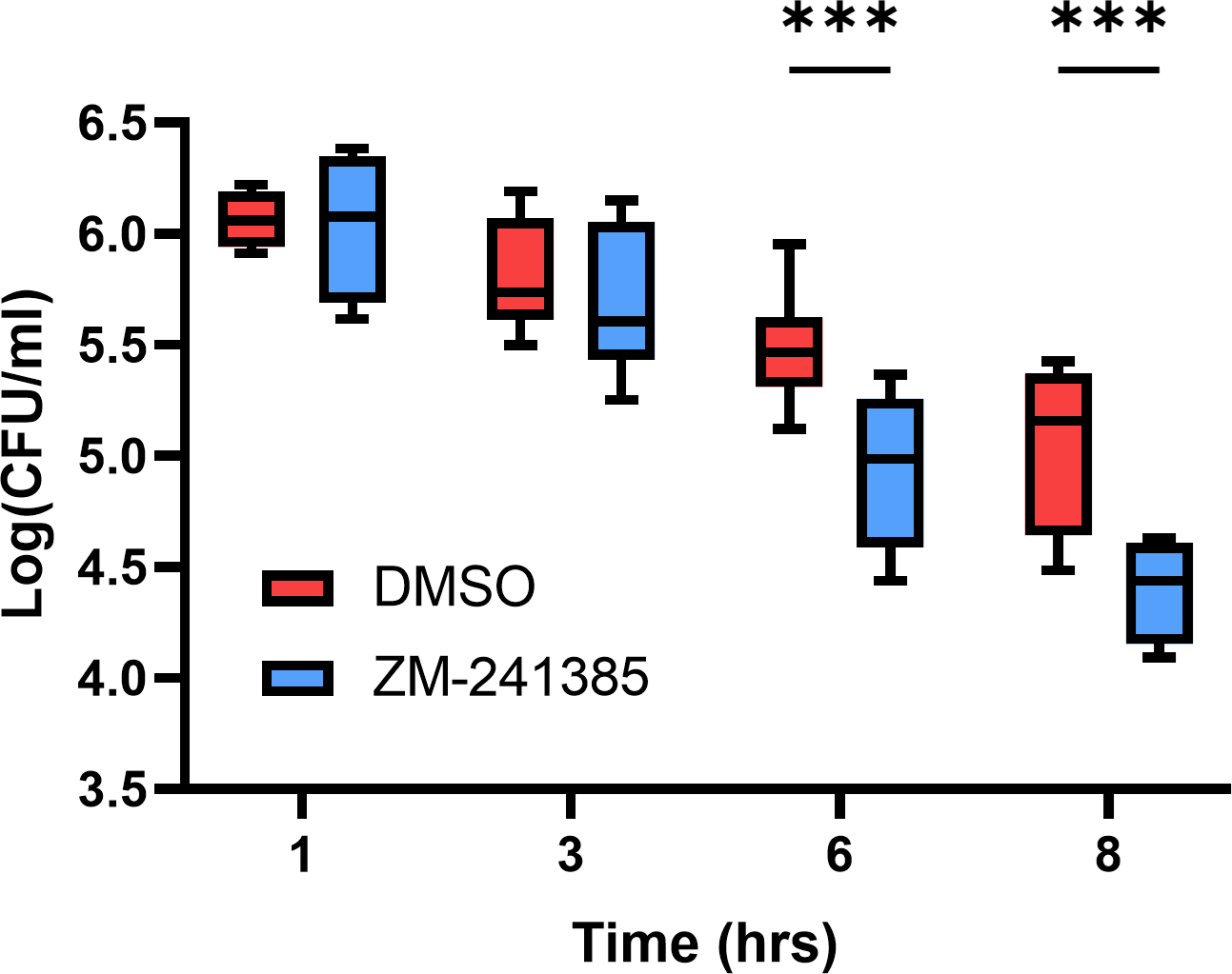
A2aR blockade with the selective inhibitor ZM-241385 significantly decreases intracellular survival of *S. aureus* within hPMN. hPMN were pre-treated with either DMSO or the A2aR inhibitor ZM-241385 for 30 min and then infected with *S. aureus* for 1 h followed by gentamicin treatment. hPMN were then lysed at 1, 3, 6, and 8 h post-gentamicin treatment and plated on TSA agar for CFU enumeration and expressed as Log_10_ CFU/mL. Statistical analysis was performed using a two-way ANOVA with Šídák’s post-test (***P < 0.001) for *n* = 4–6 independent donors.

To understand the role that A2aR plays in the intracellular survival of *S. aureus*, hPMN were pre-treated with the A2aR antagonist ZM-241385 (10 µM) 30 min prior to infection and lysed at indicated time points to enumerate intracellular *S. aureus*. A2aR blockade led to significant decreases in intracellular *S. aureus* at 6 and 8 h post-gentamicin treatment (Fig. 3). The optimal concentration of ZM-241385 was determined in initial dose-response studies (Fig. S2A), which found ZM-241385 (10 µM) to effectively reduce intracellular survival without the associated cytotoxicity observed with ZM-241385 (20 µM). While we assessed the A2aR receptor using the selective antagonist ZM-241385, it should be noted that this antagonist has some affinity for the other three adenosine receptors, mostly A2bR and least of all A3R. However, hPMN show significantly greater expression of A2aR and A3R and so it is unlikely that the inhibition of A2bR and A1R is responsible for reducing intracellular *S. aureus* (29, 30). Additionally, in this study, A3R was found to be significantly reduced in hPMN harboring intracellular *S. aureus* (Fig. 2A) further implicating a pathogenic role of A2aR activation. To definitively prove that the A2aR was responsible for this effect, the A2aR agonist, CGS 21680, was shown to significantly increase the intracellular survival of *S. aureus* within hPMN (Fig. S2B). These data imply that intracellular *S. aureus* benefits from A2aR activation which somehow facilitates its intracellular survival.

### A2aR inhibition enhances ROS production, NET formation, and IL-8 secretion in hPMN harboring intracellular *S. aureus*

**Fig 4:**
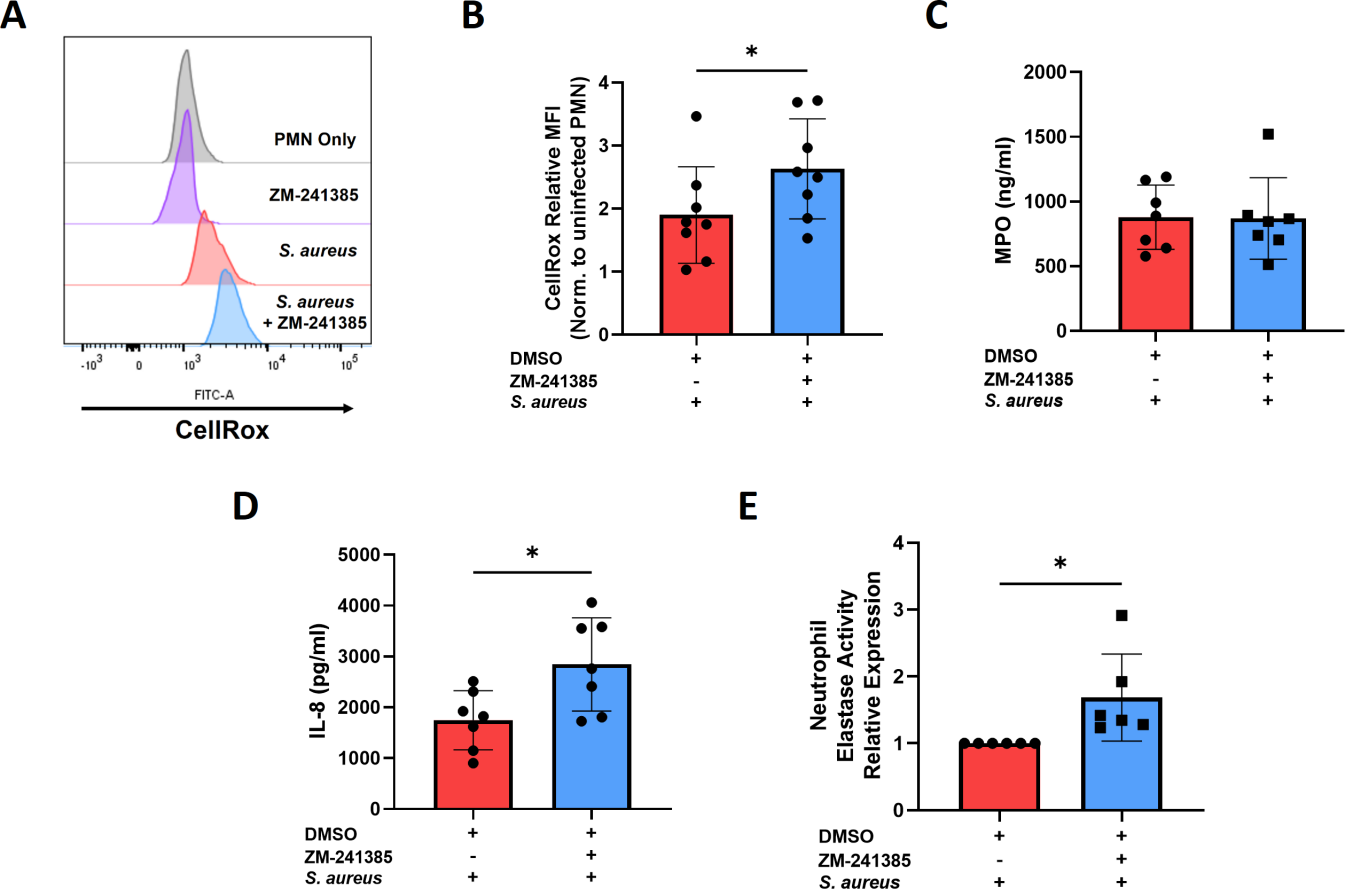
A2aR blockade with ZM-241385 leads to enhanced hPMN effector function. hPMN were infected with *S. aureus* for 1 h prior to gentamicin treatment. At 2.5 h post-gentamicin treatment, cells were incubated with CellRox for 30 min followed by flow cytometric analysis gating on single, live cells to assess ROS expression. Representative histograms are shown (**A**) alongside pooled MFI data (**B**). At 6 h post-gentamicin treatment, supernatants were collected to measure MPO (**C**) and IL-8 (**D**) secretion by ELISA. NET formation was assessed using the Abcam NETosis assay kit 3 h post-gentamicin treatment in the presence or absence of the A2aR inhibitor ZM-241385 (**E**). Statistical analysis was performed using a Wilcoxon’s test (*P < 0.05) for *n* = 6–8 independent donors.

A2aR has been shown to suppress ROS production in TNF-primed hPMN in response to fMLP as well as in LPS-treated murine PMN (31, 32). To investigate whether A2aR activation was suppressing ROS production in hPMN harboring intracellular *S. aureus*, hPMN were pre-treated with ZM-241385 prior to infection for 1 h followed by gentamicin treatment. At 2.5 h post-gentamicin treatment, hPMN were incubated with the cell-permeable ROS dye, CellRox, for 30 min and assessed by flow cytometry. ZM-241385-treated hPMN harboring intracellular *S. aureus* showed significant increases in ROS expression compared to untreated infected hPMN (Fig. 4A and B). There is evidence that A2aR agonism can suppress hPMN degranulation (33); however, MPO secretion by hPMN harboring intracellular *S. aureus* was unaffected by ZM-241285 treatment (Fig. 4C) implying A2aR does not impact degranulation in this model. ZM-241385 treatment did, however, significantly increase the production of the neutrophil-recruiting chemokine IL-8 (Fig. 4D).

Inhibition of A2aR has also been linked to increased net formation in phorbol myristate acetate (PMA)-stimulated hPMN (34). To address this, hPMN were assessed using the ABCAM NETosis assay, which uses NE as a surrogate marker for net formation. A2aR inhibition led to significant increases in NE activity over infected DMSO control indicating that the A2aR suppresses NETosis in hPMN harboring intracellular *S. aureus* (Fig. 4E).

### Inhibition of A2aR reduces the glycolytic function of hPMN harboring intracellular *S. aureus*

**Fig 5:**
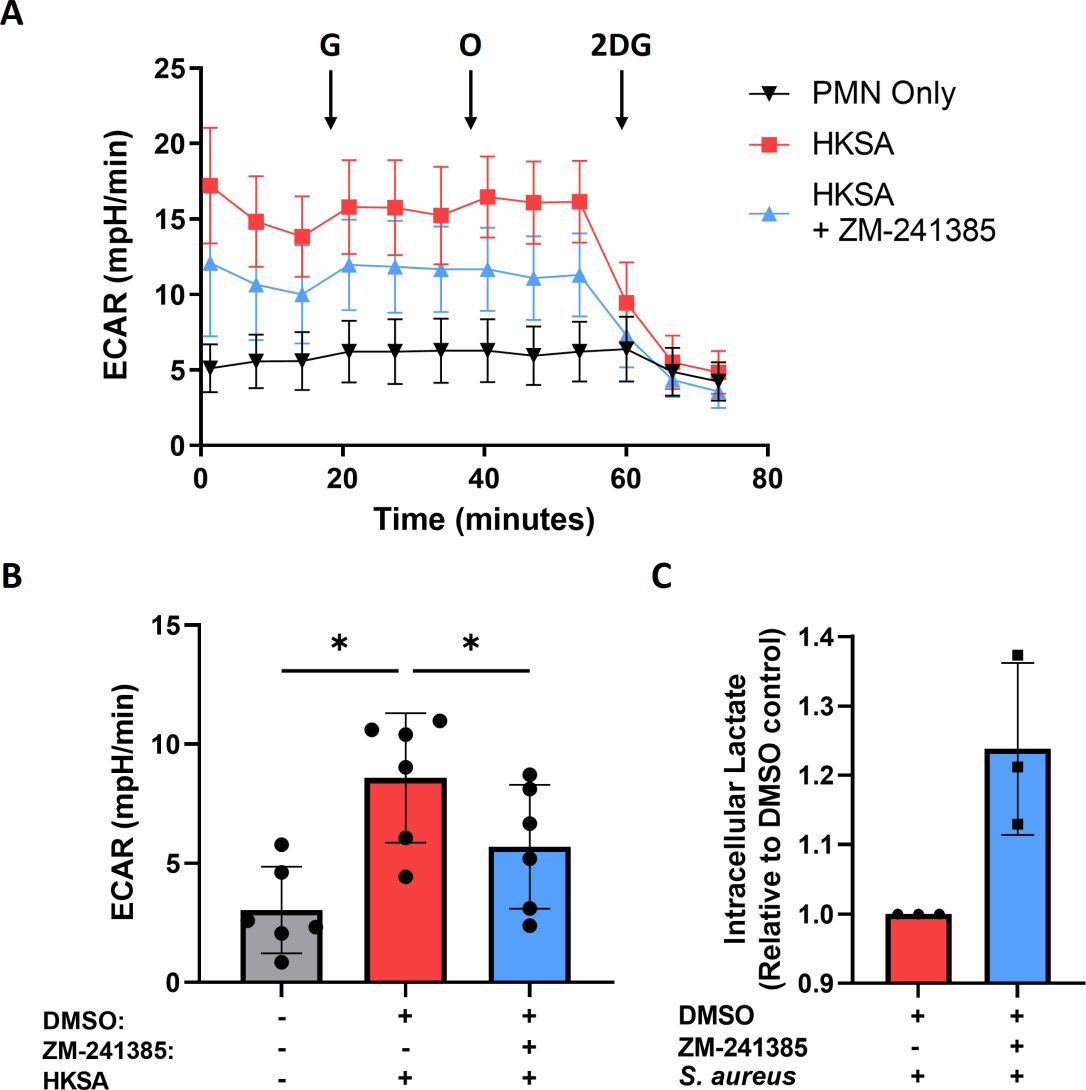
A2aR blockade with ZM-241385 significantly reduces the glycolytic activity of hPMN stimulated with heat-killed *S. aureus*. hPMN were stimulated with HKSA (2 µg/mL) for 2 h prior to metabolic analysis using an Agilent Seahorse XF/XFe analyzer to measure ECAR under basal conditions and with glucose (G), oligomycin (O), and 2DG treatment. Representative seahorse plot of unstimulated or HKSA-stimulated hPMN in the presence or absence of ZM-241385 (**A**). Basal glycolysis was calculated using ECAR values of glucose treatment minus 2DG treatment at the third interval reading (**B**). Intracellular lactate levels of *S. aureus*-infected hPMN in the presence or absence of ZM-241385 (**C**). Statistical analysis was performed using a one-way ANOVA with a Tukey’s post-test (*P < 0.05) for *n* = 6 independent donors.

Mature PMN rely heavily on glycolysis under basal conditions, with glucose uptake vital for NETosis and ROS production as hPMN decrease both in glucose-free media (35–37). To establish if the enhancement of ROS production and NETosis following ZM-241385 treatment was due to changes in PMN glycolysis, Seahorse metabolic flux analysis was employed. Extracellular acidification rate was used as a readout for glycolysis in hPMN stimulated with HKSA (2 µg/mL), in the presence or absence of ZM-241385. HKSA-stimulated hPMN exhibited a large increase in ECAR after glucose injection with negligible changes seen after oligomycin injection and complete ablation with 2DG injection (Fig. 5A). Oxidative phosphorylation is known to be metabolically dispensable for energy production in hPMN; thus, oligomycin has little effect on ECAR (35). Overall, HKSA led to a significant increase in ECAR and glycolytic capacity of hPMN, which was significantly reduced in the presence of ZM-241385 (Fig. 5B), implicating A2aR in the enhancement of hPMN glycolysis. Conversely, treatment of HKSA-stimulated hPMN with adenosine resulted in enhanced glycolysis (Fig. S3), further implicating the A2aR in the enhancement of hPMN glycolysis. ZM-241385 also increased the levels of intracellular lactate produced during *S. aureus* intracellular survival in hPMN (Fig. 5C). Given that lactate is known to inhibit cellular glycolysis (38, 39), this suggests that A2aR enhances the export of intracellular lactate to maintain glycolytic function.

### Inhibition of A2aR enhances the PPP in hPMN harboring intracellular *S. aureus* resulting in enhanced ROS production

**Fig 6:**
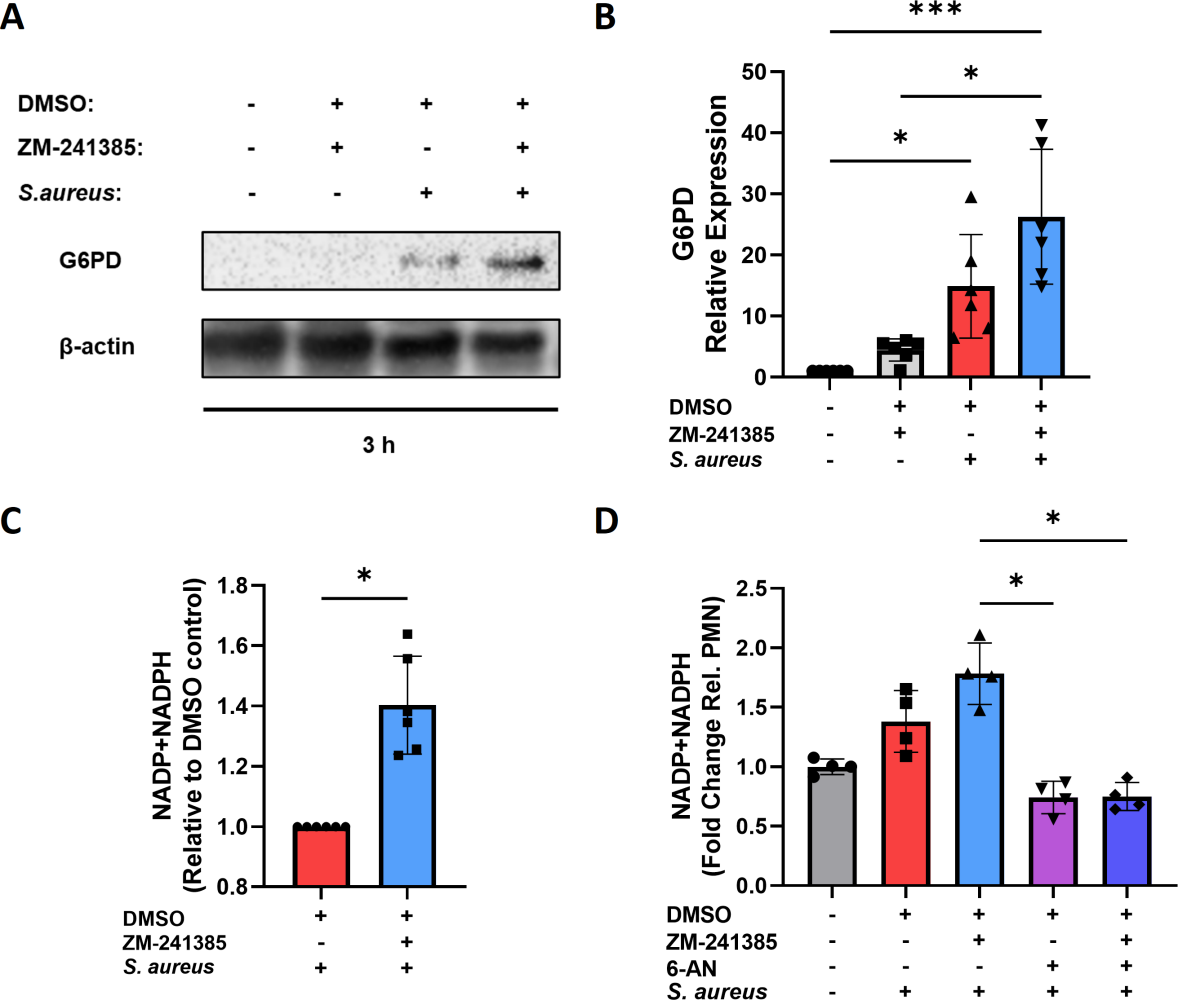
Inhibition of A2aR in hPMN harboring intracellular *S. aureus* enhances the PPP. hPMN were pre-treated with either DMSO or the A2aR inhibitor ZM-241385 for 30 min and then infected with *S. aureus* for 1 h followed by gentamicin treatment. At 3 h post-gentamicin treatment, G6PD levels were measured using western blotting with a representative blot shown (A) alongside pooled densitometric analysis (B). At 1 h post-gentamicin treatment, NADP + NADPH levels of hPMN harboring intracellular *S. aureus* were assessed in the presence or absence of ZM-241385 with or without 6-AN treatment using an NADP/NADPH quantitation kit (C and D). Statistical analysis was performed using a paired Student’s *t* test for single comparison, and a one- or two-way ANOVA for multiple comparisons (*P < 0.05, ***P < 0.001, and ****P < 0.0001) for *n* = 6 independent donors.

The PPP is a critical metabolic pathway involved in the generation of NADPH and ROS via the NADPH oxidase. PMA-stimulated hPMN have been shown to shunt glycolytic intermediates into the PPP to enhance NADPH production (40). As A2aR inhibition in *S. aureus*-stimulated hPMN leads to enhanced ROS production yet significantly decreased glycolysis, it was hypothesized that the PPP may be involved. To investigate this, hPMN were infected with *S. aureus* in the presence or absence of ZM-241385 and lysed 3 h post-gentamicin treatment for western blotting. Glucose-6 phosphate dehydrogenase (G6PD), the rate-limiting step in oxidative PPP, was found to be significantly upregulated in hPMN harboring intracellular *S. aureus* compared to uninfected hPMN, while A2aR blockade led to a further increase (Fig. 6A and B), suggesting upregulation of the PPP. To confirm this, combined NADP + NADPH levels within *S. aureus* infected hPMN in the presence or absence of ZM-241385 were assessed using an NADP/NADPH quantitation kit, which quantifies both metabolites simultaneously by converting NADP into NADPH prior to NADPH quantification. A2aR blockade led to significant increases in NADP + NADPH within hPMN harboring intracellular *S. aureus* relative to infected DMSO control (Fig. 6C). This result was validated using the PPP inhibitor 6-aminonicatinamide (6-AN), which showed substantial reductions in NADP + NAPDH of 6-AN-treated and infected hPMN regardless of A2aR blockade (Fig. 6D). This is as expected as 6-AN inhibits G6PD, which, in this study, is enhanced by A2aR blockade, preventing the generation of NADP + NADPH by the PPP.

**Fig 7:**
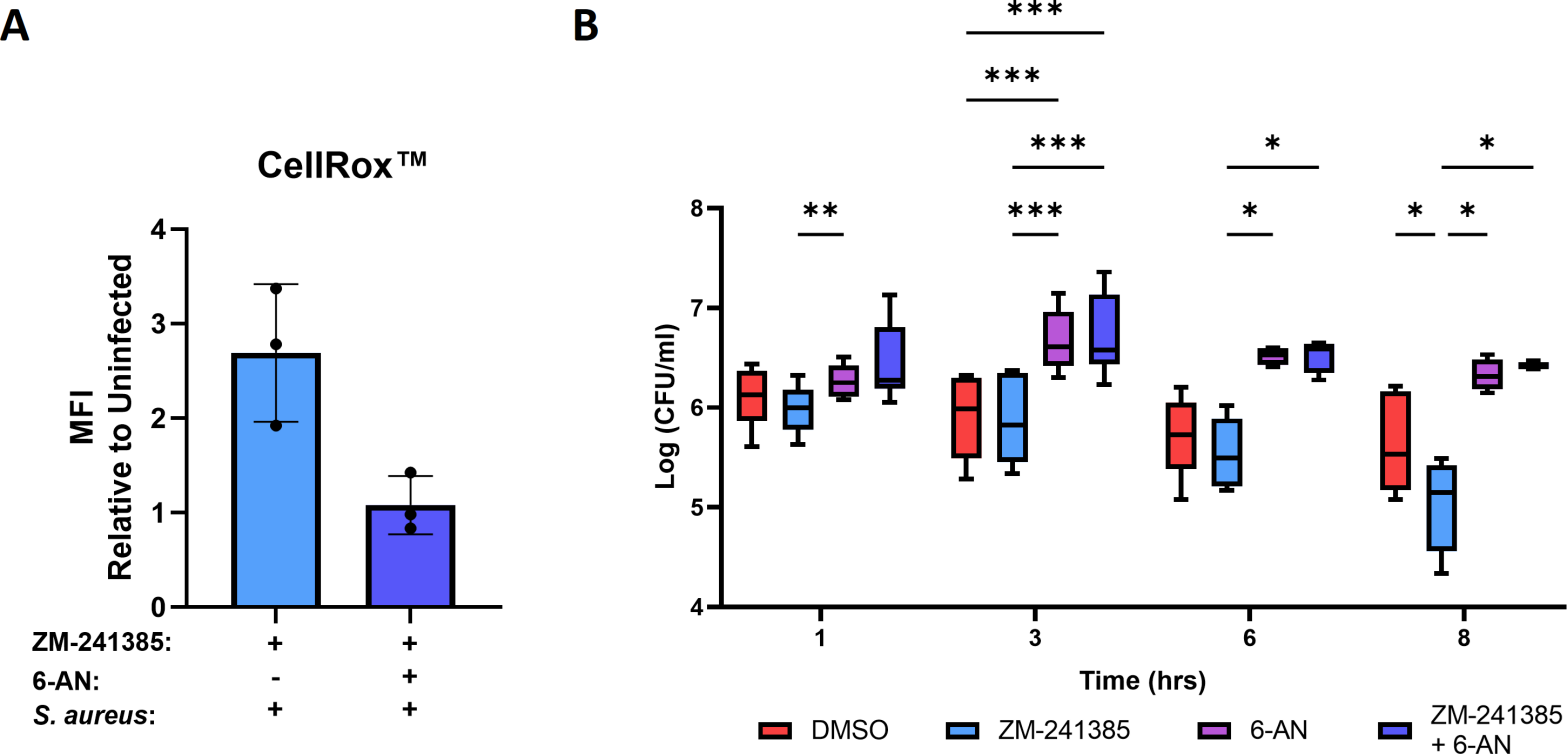
Inhibition of the PPP in hPMN reduces intracellular ROS and significantly enhances intracellular survival of *S. aureus*. hPMN were pre-treated with either DMSO or ZM-241385 for 30 min with or without 6-AN (500 µM) and then infected with *S. aureus* for 1 h followed by gentamicin treatment. ROS production was then assessed at 3 h (**A**) post-gentamicin treatment. Additionally, hPMN were lysed at 1, 3, 6, and 8 h post-gentamicin treatment, serially diluted, and plated on TSA plates for CFU enumeration (**B**). Statistical analysis was performed using a paired Student’s *t* test for single comparison, and a one- or two-way ANOVA for multiple comparisons (*P < 0.05, ***P < 0.001, and ****P < 0.0001) for *n* = 4–6 independent donors.

To highlight the importance of the PPP during intracellular survival of *S. aureus* in hPMN, intracellular ROS production was assessed using CellRox in ZM-241385-treated hPMN harboring intracellular *S. aureus* with or without 6-AN. 6-AN treatment reduced intracellular ROS in ZM-241385-treated hPMN harboring intracellular *S. aureus* (Fig. 7A). Finally, to determine the importance of PPP in controlling intracellular survival of *S. aureus* within hPMN, infected hPMN were treated with DMSO or ZM-241385 with or without 6-AN (Fig. 7B). Inhibition of PPP significantly enhanced intracellular burden in both ZM-241385-treated and untreated cells, highlighting the importance of a fully functional PPP to limit the intracellular burden of *S. aureus*.

## DISCUSSION

Intracellular survival of *S. aureus* within phagocytes is known to contribute to disease severity and dissemination whilst also limiting antibiotic efficacy (4, 11, 41). While some insights into the mechanism of intracellular survival exist, our understanding of the process is limited. To develop novel therapeutics and vaccines, it is critical that we understand the intracellular lifestyle of *S. aureus*.

To understand how hPMN harboring intracellular *S. aureus* are reprogrammed, we employed gene expression analysis. Pathway analysis revealed hPMN harboring intracellular *S. aureus* upregulated pro-inflammatory mediators that are consistent with bacterial infection, in addition to the autophagy regulator p62, several anti-apoptotic genes, and the p53-induced *cdkn1a*. This is consistent with previous studies that showed that p53 induced DRAM expression to enhance autophagy and decrease hPMN apoptosis, leading to enhanced intracellular survival and prolonged hPMN survival (16). Additionally, pathway analysis highlighted the upregulation of the anti-inflammatory A2aR and several downstream mediators of A2aR-induced immunosuppression. A2aR activation can delay neutrophil aging and cell death while polarizing PMN to the anti-inflammatory N2 phenotype *in vitro* (42) and, in theory, would benefit the survival of *S. aureus* intracellularly. In our model, A2aR blockade decreased the intracellular survival of *S. aureus* in hPMN and was associated with enhanced ROS production and NET formation. Thus, it appears that *S. aureus* may utilize this pathway to suppress hPMN effector functions to survive intracellularly.

Autophagy has been shown to facilitate *S. aureus* intracellular survival (4, 16, 17). How autophagy relates to A2aR signaling in the context of *S. aureus* intracellular survival, however, remains to be established. A2aR activation of LPS-stimulated murine PMN has previously been shown to inhibit autophagy by suppressing ROS production and JNK activation while simultaneously activating AKT signaling to further restrict autophagy (31). Inhibition of autophagy is typically associated with decreased intracellular survival of *S. aureus* (16). However, here we show that A2aR activation enhances intracellular survival of *S. aureus* in hPMN with A2aR blockade leading to decreases in intracellular burden. This does not appear to be associated with any significant changes in LC3-II accumulation (Fig. S4), suggesting A2aR in this context does not impact autophagic flux. Potentially, intracellular *S. aureus* induces and disrupts autophagy prior to A2aR activation, severing the inhibitory link that A2aR has on autophagy while retaining the immunosuppressive role of A2aR to protect its intracellular niche.

The high-affinity A2aR and low-affinity A2bR play important roles in many diseases such as cancer, neurodegenerative disorders, and sepsis (43–45). A2aR is basally expressed in hPMN, which can be enhanced by both LPS and T_h_1 cytokines, suggesting a regulatory role during the immune response (30). However, hPMN from septic patients have significantly higher expression of A2aR compared to healthy controls, and in a murine septic model, treatment with ZM-241385 can boost survival rates by enhancing bacterial clearance (42, 46). In this murine sepsis model, A2aR was shown to induce Tregs, which suppress PMN effector function thus dampening and prolonging the dysregulated inflammatory response associated with sepsis (45). In our study, we show for the first time that adenosine signaling through A2aR can support *S. aureus* survival by interfering with the PPP in hPMN, suppressing ROS production that would otherwise kill intracellular *S. aureus*. To our knowledge, this is the first study showing that A2aR activation can suppress the PPP, a pathway that appears to be critical in the elimination of intracellular *S. aureus*. In addition to suppressing PMN function, it is possible that *S. aureus* may limit the adaptive immune response through the production of NET-derived adenosine to induce Treg cells, further suppressing PMN as well as other critical immune protective responses. *S. aureus*-derived adenosine has been shown to impact the immune response by limiting Th1/Th17-inducing cytokines, which would otherwise confer protection against recurrent infection (47). Treatment with ZM-241385, therefore, potentially represents a viable option for treating *S. aureus* infection and sepsis.

PMN are largely glycolytic but can shunt glycolytic intermediates into other metabolic pathways such as the PPP in response to stimuli such as PMA, for ROS production and NETosis (37, 40). In this study, hPMN activated in response to *S. aureus* led to a significant increase in glycolysis compared to unstimulated hPMN. However, under A2aR blockade, hPMN showed decreased glycolytic capacity despite A2aR inhibition leading to enhanced effector function and decreased intracellular survival. Additionally, A2aR inhibition substantially enhanced intracellular lactate implying A2aR may be involved in lactate export, which would otherwise inhibit hPMN glycolysis. Few studies have examined the role A2aR plays in host metabolism, but limited evidence shows that A2aR deletion can significantly decrease glycolysis in endothelial cells (28).

As activated PMN can shunt the glycolytic intermediate glucose-6-phosphate into the PPP to enhance ROS and NET production (35), we assessed the PPP to determine if A2aR blockade created a metabolic switch from glycolysis to PPP to fuel the NAPDH oxidase system. PMA-treated hPMN shunt glycolytic intermediates into the PPP to increase the necessary NADPH pool fueling the NADPH oxidase complex leading to enhanced ROS production and a decrease in lower glycolytic outputs (40). However, in our study, we found significant increases in G6PD, the rate-limiting enzyme of the PPP, in hPMN harboring intracellular *S. aureus*, which was enhanced by the A2aR blockade. This would explain the increase in NADP + NADPH levels under A2aR blockade as G6PD enhances NADPH output. In a previous study, Britt et al. found in unstimulated and PMA-stimulated HL-60s that G6PD levels were unchanged and that NADPH was rapidly consumed and replenished by NADPH cycling by reversed upper glycolysis (40). This difference is potentially due to many factors such as stimulus (PMA vs live bacteria), time points (30 min vs 4 h), or primary cells vs cell line but our study suggests *S. aureus*-infected hPMN show enhanced NADPH production via G6PD in comparison to PMA stimulation. Additionally, ZM-241385 enhanced the NADP + NADPH content of infected hPMN relative to DMSO controls implicating an increase in the PPP when A2aR is blocked. To our knowledge, this is the first time A2aR has been associated with hPMN metabolism representing an important interaction that enhances glycolysis while limiting the PPP and reducing NADP + NADPH levels that would otherwise fuel the NADPH oxidase system.

**Fig 8:**
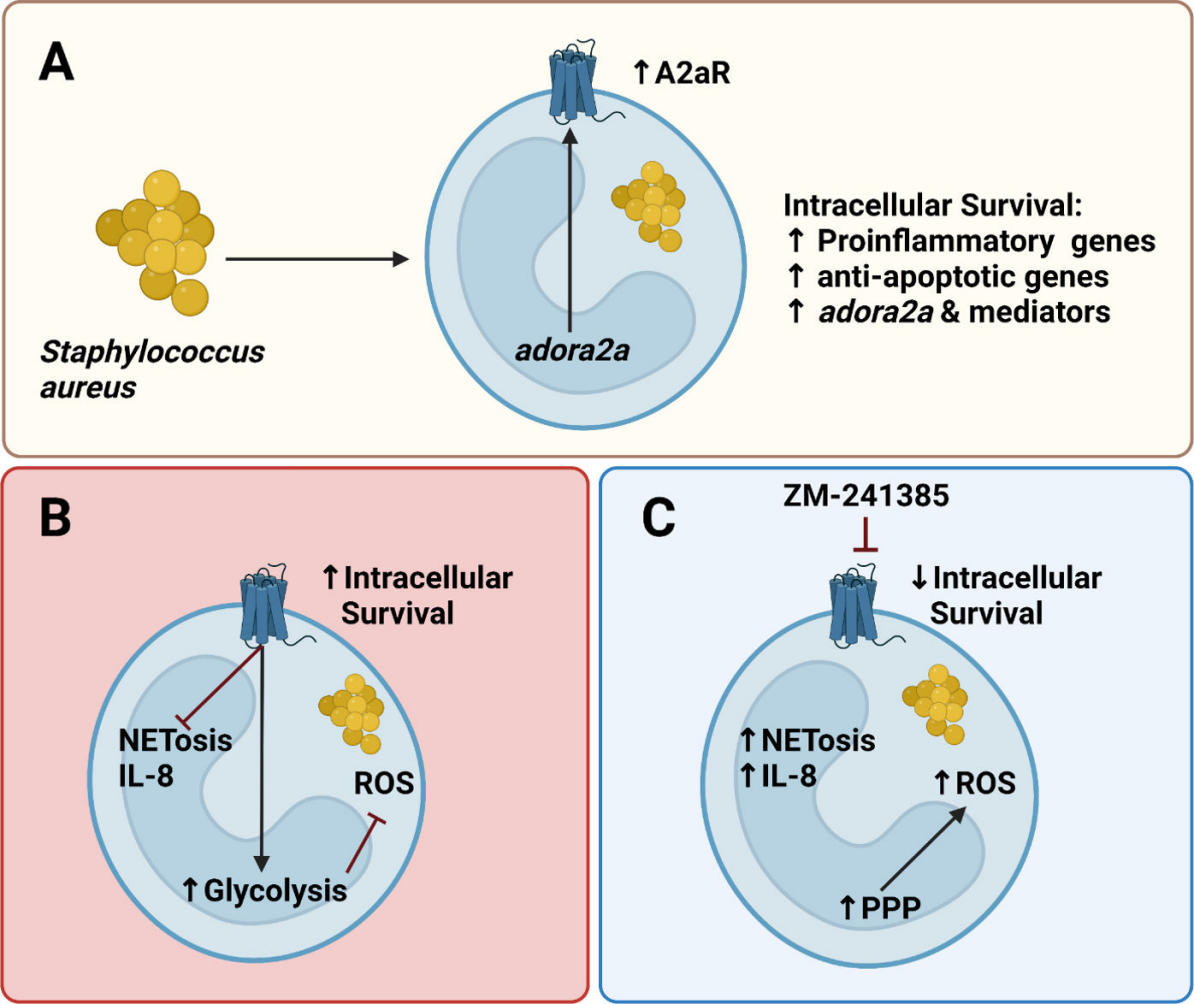
Schematic overview of *S. aureus*-induced A2aR expression and its effect on intracellular survival. Intracellular *S. aureus* leads to the upregulation of several pro-inflammatory and anti-apoptotic genes as well as upregulating the adenosine receptor *adora2a* and several associated downstream mediators (**A**). A2aR activations lead to a suppressed hPMN phenotype leading to decreased NETs and IL-8 while also increasing hPMN glycolysis and reducing PPP, which limits ROS production and increases intracellular survival of *S. aureus* (**B**). A2aR inhibition with ZM-241385 leads to enhanced NET formation and IL-8, while also diverting hPMN glycolysis to the PPP, enhancing ROS production, and reducing the intracellular survival of *S. aureus* (**C**).

In summary, we propose a novel mechanism by which *S. aureus* restricts hPMN effector functions to facilitate its intracellular survival. Upon *S. aureus* exposure, hPMN upregulate A2aR (Fig. 8A), which *S. aureus* manipulates to enhance hPMN glycolysis over PPP, thus restricting NADPH levels that would otherwise fuel ROS production to eliminate intracellular *S. aureus* (Fig. 8B). We show that A2aR activation restricts the PPP and enhances host glycolysis in hPMN. Additionally, we show that A2aR activation limits NETosis and IL-8, thus providing further evidence of *S. aureus* suppressing the local immune response for its own gain. By intervening with the A2aR antagonist ZM-241385, hPMN functionality can be effectively restored to limit intracellular survival by enhancing the PPP, ROS production, and NETosis (Fig. 8C). Taken together, our data highlight the importance of the A2aR receptor as well as its potential as a therapeutic target in *S. aureus* infection to limit intracellular survival. A limitation of the study is that it did not identify the specific source of adenosine. *S. aureus* is known to produce the AdsA enzyme, which can form adenosine in concert with Nuc from hPMN NETs (48). However, there is the potential that host-derived adenosine from lysed cells is also a potential source. hPMN harboring intracellular *S. aureus* were found to significantly increase CD39 surface expression (Fig. S5), which is an ectoenzyme that converts ATP into AMP that can be further processed into adenosine by CD73 (33). So, it is likely that both the host and *S. aureus* itself contribute to elevated levels of extracellular adenosine, which ultimately signals through the A2aR to enhance intracellular survival (48). Future studies should aim to address this outstanding question as well as employ metabolomic approaches to gain a full metabolic overview of hPMN harboring intracellular *S. aureus*.

## SUPPLEMENTAL MATERIAL

Supplemental Material mbio.02571-23-s0001.pdf Supplemental material showing dose response to A2aR inhibitor as well as examining the role of autophagy in A2aR activation and highlighting host derived generation of adenosine.

ASM does not own the copyrights to Supplemental Material that may be linked to, or accessed through, an article. The authors have granted ASM a non-exclusive, world-wide license to publish the Supplemental Material files. Please contact the corresponding author directly for reuse.
