# Supplementary material for: Staphylococcus aureus suppresses the pentose phosphate pathway in human neutrophils via the adenosine receptor A2aR to enhance intracellular survival
Source: mBio. 2023 Dec 18;15(1):e02571-23. doi: 10.1128/mbio.02571-23 (PMC10790693; doi:10.1128/mbio.02571-23)
Supplement: Supplemental Material — showing dose response to A2aR inhibitor as well as examining the role of autophagy in A2aR activation and highlighting host derived generation of adenosine. [file mbio.02571-23-s0001.pdf]

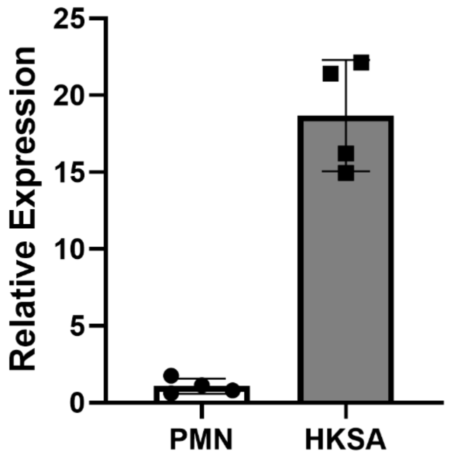

Supplemental Figure 1: Heat killed *S. aureus* (HKSA) stimulated hPMN upregulate A2aR expression.

hPMN were stimulated with HKSA (2 µg/ml) for 4 h and then lysed for RNA. RT-PCR assessment of *adora2a* was conducted using the ΔΔCT method and *actb* as a housekeeping gene (A). Statistical analysis was preformed using a Wilcoxon's test (**P* <0.05) for n = 4 independent donors.

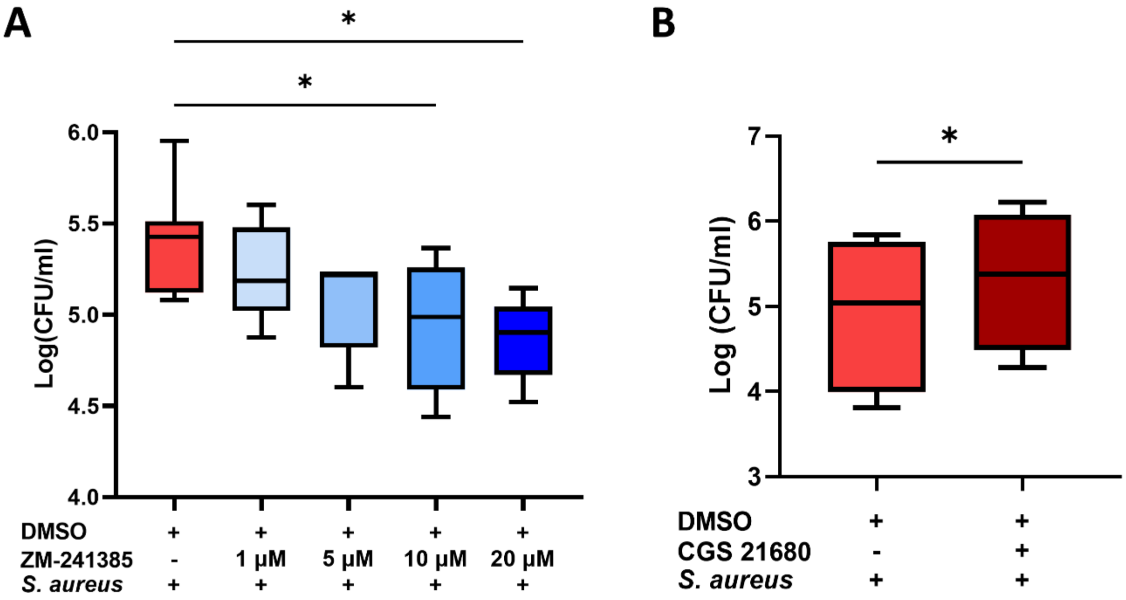

Supplemental Figure 2: Inhibition of the A2aR with ZM-241385 significantly decreases intracellular survival of *S. aureus* in hPMN whilst activation of A2aR with CGS 21680 significantly increases intracellular burden.

hPMN were pre-treated with either DMSO, the A2aR inhibitor ZM-241385 (A) or the A2aR agonist CGS 21680 (B) (1 µM) for 30 mins and then infected *S. aureus* for 1 h followed by gentamicin treatment. hPMN were then lysed at 6 h post gentamicin treatment and plated on TSA agar for CFU enumeration and expressed as Log_{10} CFU/ml. Statistical analysis was preformed using a one-way ANOVA with Tukey post-test (****P* <0.001) for n = 4-6 independent donors.

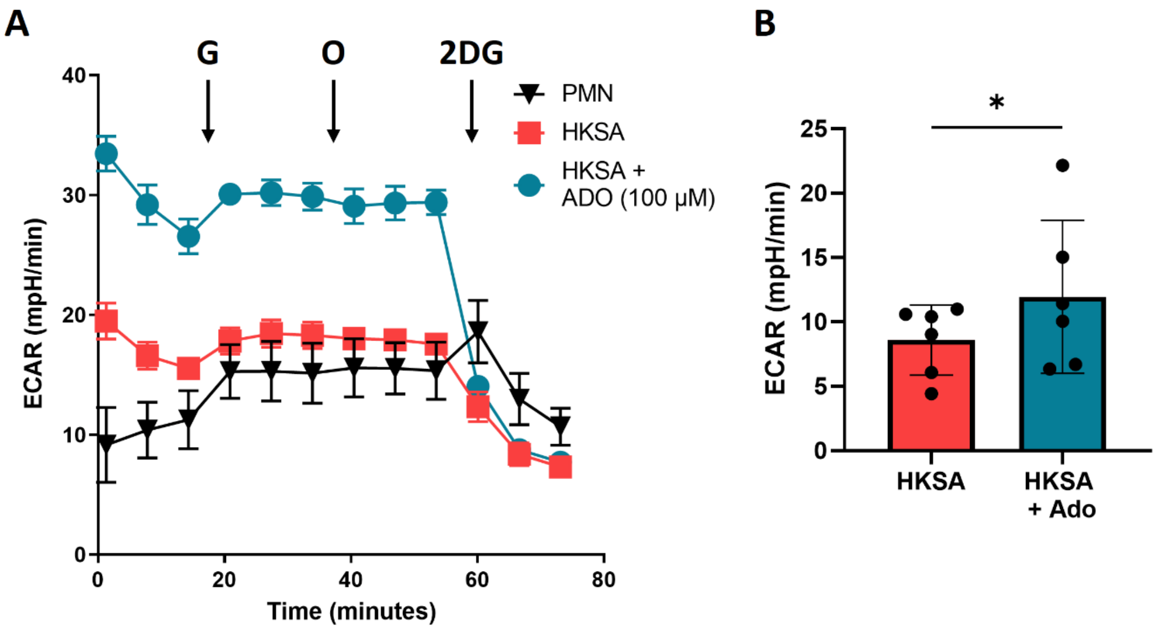

Supplemental Figure 3: Treatment of HKSA-treated hPMN enhances host glycolysis.

hPMN were stimulated with HKSA (2 μg/ml) plus or minus adenosine (100uM) for 2 h prior to metabolic analysis using an Agilent Seahorse XF/XFe analyser to measure ECAR under basal conditions and with glucose (G), Oligomycin (O) and 2-deoxyglucose (2DG) treatment. Representative seahorse plot of ECAR of unstimulated or HKSA stimulated hPMN pre-treated with DMSO or Adenosine (ADO, 100 μM) as a readout for glycolysis (A). Basal glycolysis was calculated using ECAR values of glucose treatment minus 2DG treatment at the third interval reading (B). Statistical analysis was preformed using a one-way ANOVA with a Tukey post-test (**P* <0.05) for n = 6 independent donors.

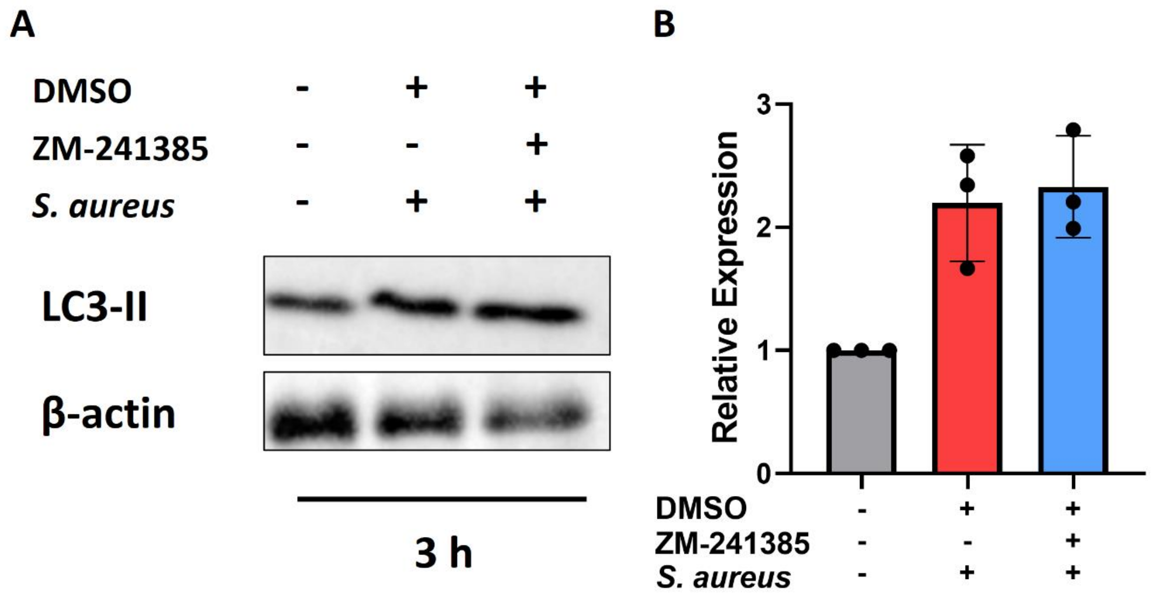

Supplemental Figure 4: A2aR inhibition does not impact LC3-II accumulation in hPMN harbouring intracellular *S. aureus*.

hPMN were pre-treated with either vehicle or ZM-241385 for 30 mins and then infected for 1 h followed by gentamicin treatment. 3 h post gentamicin treatment cells were lysed for western blotting and LC3-II levels assessed. Statistical analysis was preformed using a one-way ANOVA with a Tukey post-test for n = 3 independent donors.

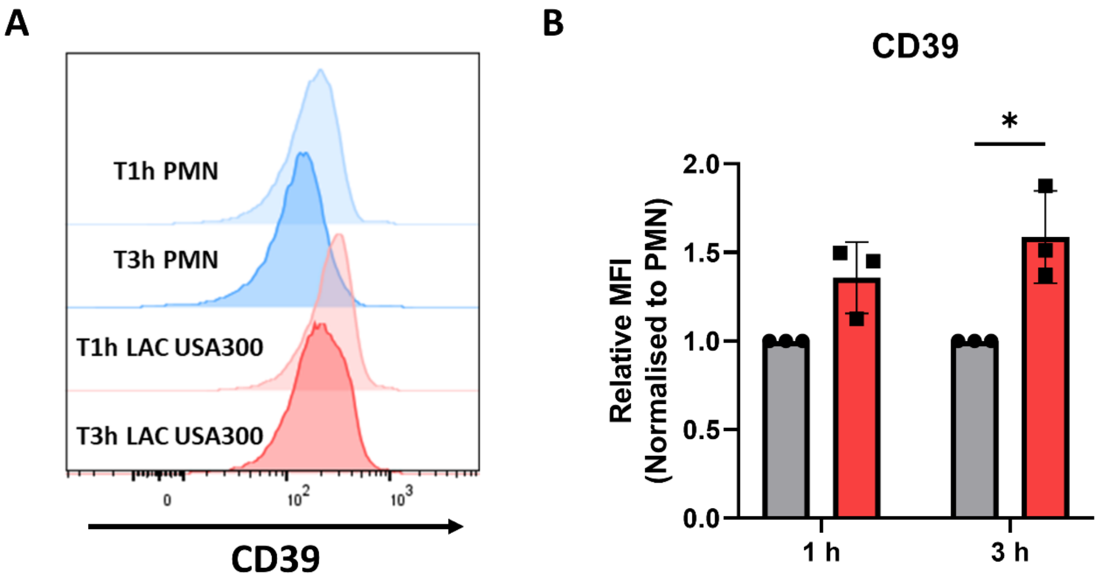

Supplemental Figure 5: hPMN harbouring intracellular *S. aureus* significantly increase surface expression of CD39, an adenosine promoting ectoenzyme.

hPMN were infected with *S. aureus* for 1 h prior to gentamicin treatment and assessed using flow cytometry 1 h and 3 h post gentamicin treatment. Flow cytometric assessment of CD39 was conducted by gating on single, live cells to obtain representative histograms (A) and MFI relative to uninfected hPMN (B). Statistical analysis was conducted using a two-way ANOVA with Šídák's post-test (*$P < 0.05$) for n = 3 independent donors.

Uncropped Western Blots

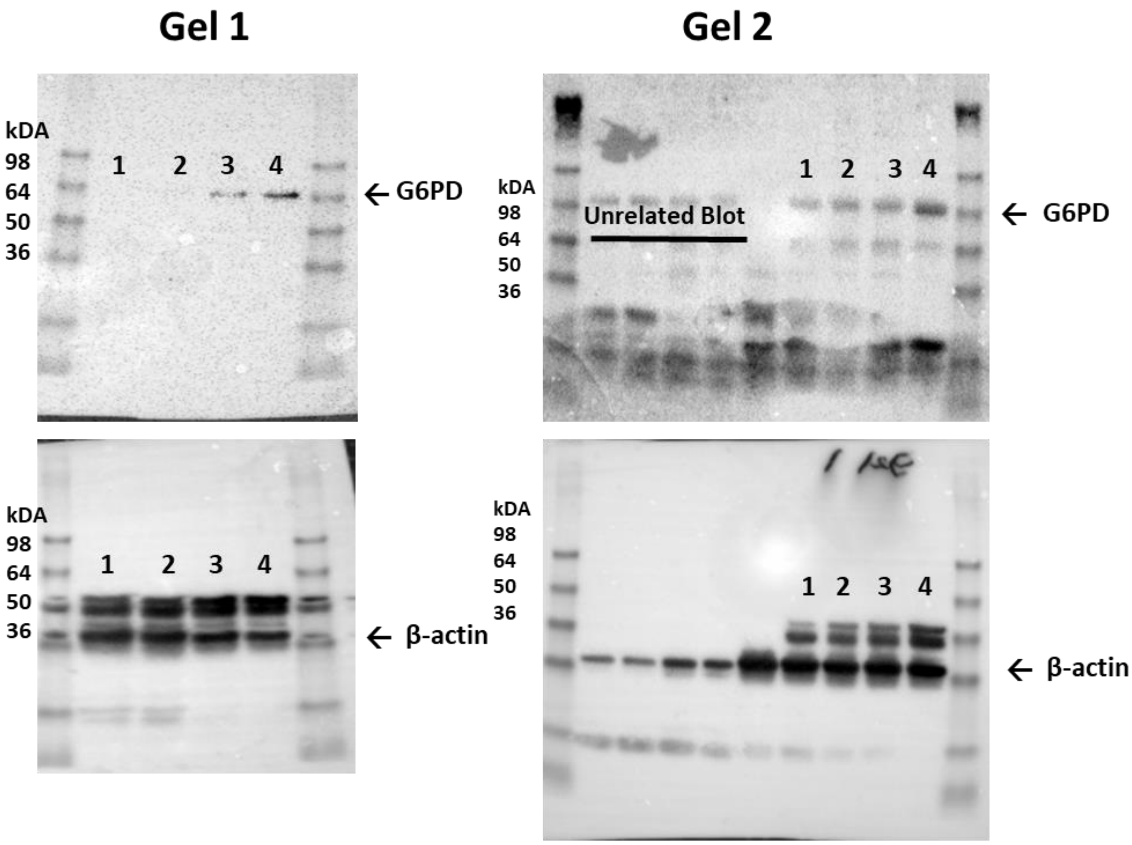

1. Uninfected PMN   2. ZM-241385 treated PMN   3. PMN + S. aureus   4. PMN + S. aureus + ZM-241385

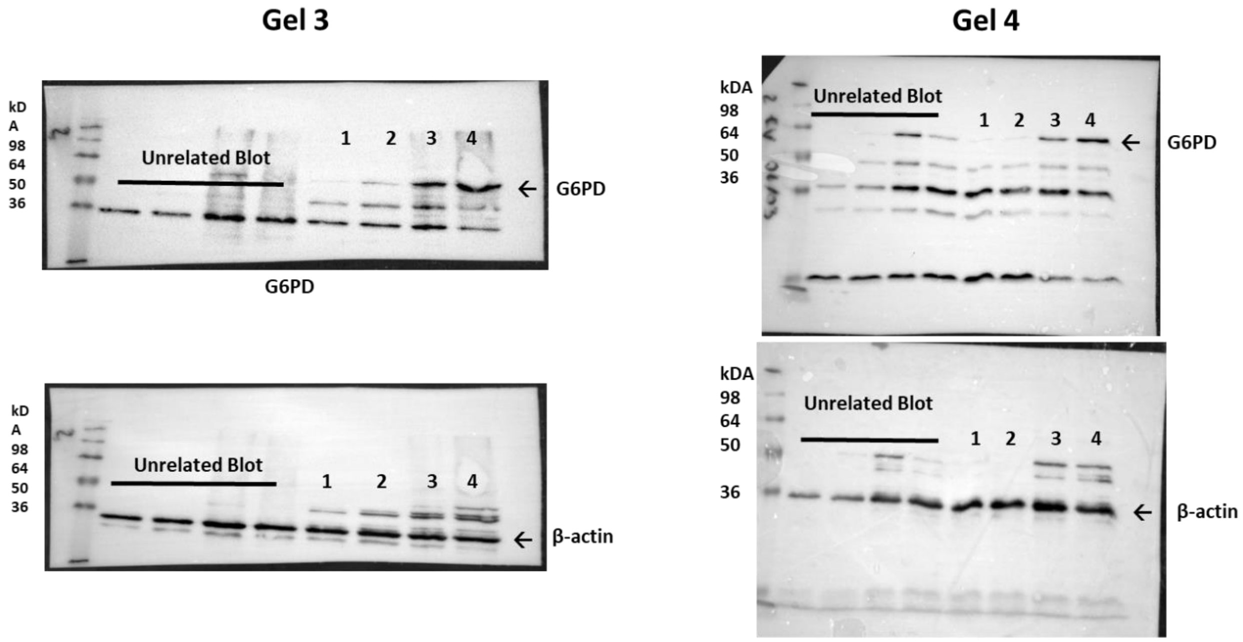

1. Uninfected	2. ZM-241385 treated	3. PMN + S. aureus	4. PMN + S. aureus + ZM-241385

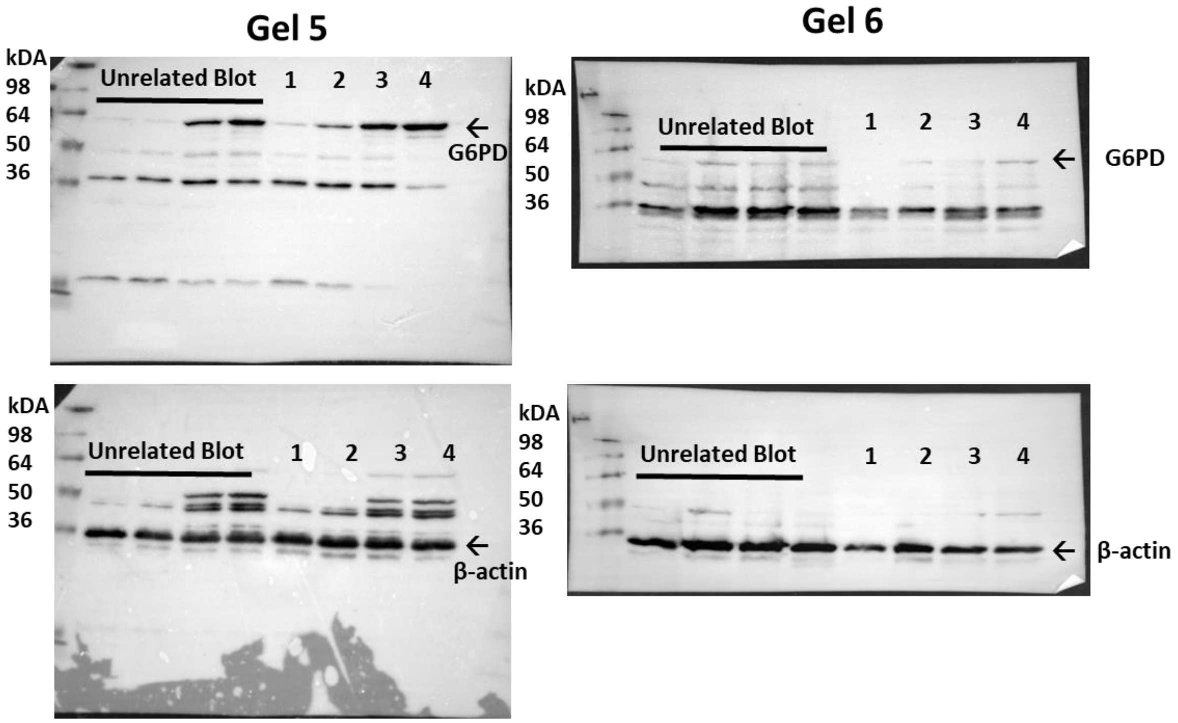

1. Uninfected PMN 2. ZM-241385 treated PMN 3. PMN + *S. aureus* 4. PMN + *S. aureus* + ZM-241385
